# Intratumoral KIT mutational heterogeneity and recurrent KIT/ PDGFRA mutations in KIT/PDGFRA wild-type gastrointestinal stromal tumors

**DOI:** 10.18632/oncotarget.7148

**Published:** 2016-02-02

**Authors:** Jing Gao, Jian Li, Yanyan Li, Zhongwu Li, Jifang Gong, Jian Wu, Na Liu, Bin Dong, Changsong Qi, Jie Li, Lin Shen

**Affiliations:** ^1^ Department of Gastrointestinal Oncology, Key laboratory of Carcinogenesis and Translational Research (Ministry of Education/Beijing), Peking University Cancer Hospital and Institute, Beijing, China; ^2^ Department of Pathology, Key Laboratory of Carcinogenesis and Translational Research (Ministry of Education/Beijing), Peking University Cancer Hospital and Institute, Beijing, China; ^3^ MyGenostics Inc. Beijing, China

**Keywords:** wild-type GISTs, KIT/PDGFRA mutation, intratumoral heterogeneity, next-generation sequencing, imatinib

## Abstract

**Objective:**

Gastrointestinal stromal tumors (GISTs) with no mutations in exons 9, 11, 13, and 17 of the *KIT* gene and exons 12, and 18 of the *PDGFRA* gene were defined as KIT/PDGFRA wild-type and they accounted for ~15–20% of GISTs. However, some KIT/PDGFRA wild-type GISTs with KIT mutations in other exons were occasionally reported. We therefore assessed GISTs to understand the whole genomic genotypes of *KIT* or *PDGFRA* genes in KIT/PDGFRA wild-type GISTs.

**Methods:**

A cohort of 185 KIT/PDGFRA wild-type GISTs from 1,080 cases was retrospectively assessed. Thirty-nine patients were excluded due to insufficiency of genomic DNA data or failure of library preparation, and 146 patients were analyzed by targeted next-generation sequencing (NGS) followed by validation.

**Results:**

For hot spots in *KIT* and *PDGFRA* genes, 23 out of 146 KIT/PDGFRA wild-type cases carried mutations according to NGS; there were 19 KIT mutations and 4 PDGFRA mutations, and these were exclusive. Intratumoral KIT mutational heterogeneity was observed in 4 of 19 samples which potentially triggered mechanisms of polyclonal evolution and metastasis and drug sensitivity. Eleven patients treated with imatinib were evaluable for clinical response, and 2 of 3 patients with KIT mutations achieved partial response (PR), while only 1 of 8 patients without KIT mutations reached PR.

**Conclusion:**

NGS had the potential property to identify partial mutant tumors from a subset of GISTs regarded as KIT/PDGFRA wild-type tumors using Sanger sequencing, and provided a better understanding of KIT/PDGFRA genotypes as well as identified patients eligible for imatinib therapy.

## INTRODUCTION

Gastrointestinal stromal tumors (GISTs) are the most common mesenchymal tumor of gastrointestinal (GI) tract, but they are relatively uncommon ~10–20 per GISTs per million people [1]. The major mechanisms of tumorigenesis of GISTs are oncogenic mutations of the *KIT* or *PDGFRA* genes and these account for 80–90% of GISTs [2]. According to the National Comprehensive Cancer Network guidelines, GISTs with no mutations in exons 9, 11, 13, and 17 of the *KIT* gene and exons 12, and 18 of the *PDGFRA* gene are defined as KIT/PDGFRA wild-type GISTs, and they represent 15–20% of GISTs [3]. However, pathogenic mechanisms and molecular characteristics of KIT/PDGFRA wild-type GISTs are poorly understood. Recently, frequent succinate dehydrogenase (SDH) mutations were identified in KIT/PDGFRA wild-type GISTs, especially in pediatric patients, which was considered a subtype of GISTs [4, 5].

Imatinib mesylate (imatinib) is the only first-line drug for GIST treatment and efficacy depends on *KIT* or *PDGFRA* genotypes [6, 7]. Drug response in KIT/PDGFRA wild-type patients is poor and data show that 70% of these patients are resistant to imatinib. Thus, ~30% of KIT/PDGFRA wild-type patients may benefit from imatinib, suggesting susceptible factors in wild-type individuals or that some mutations are not detected with current sequencing methods.

Several studies have explored possible mechanisms of imatinib resistance and GIST pathogenesis. Miranda's group reported that KRAS and BRAF mutations existed in GIST patients that these predicted imatinib resistant in *in vitro* experiments [8]; however, no KRAS mutation was found in a cohort of 514 cases [9]. PTEN-deficient expression and PI3K/AKT pathway activation were shown to be important to imatinib resistance [10, 11]. A subset GISTs tested *in vitro* had KIT mutations in exon 8, and these cells were sensitive to imatinib [12]. To explore unknown mutations and possible pathogenic mechanisms of KIT/PDGFRA wild-type GISTs, we sequenced *KIT* and *PDGFRA* genes and critical molecules downstream of these genes using targeted next-generation sequencing (NGS).

## RESULTS

### Patient characteristics

We studied 146 KIT/PDGFRA wild-type patients and these data appear in Table 1. All patients had records of primary tumor sites, tumor sizes and mitosis, however, CD117, DOG-1, and CD34 expressions were collected from 139, 93, and 132 patients, respectively. Among 146 patients, 12 patients received imatinib palliative treatment after diagnosis, 2 patients received imatinib neoadjuvant therapy followed by surgery, 18 patients received imatinib adjuvant therapy after surgery, 2 patients received sunitinib palliative treatment when diagnosis, and the rest 112 patients received surgery alone or no any treatment when diagnosis.

**Table 1:** Characteristics of patients

Characteristics	No. of patients (%)
Sex	
Male	69 (47.3)
Female	77 (52.7)
Age (years)	
Median	52
Range	16–78
Primary sites	
Stomach	56 (38.3)
Small bowel	41 (28.1)
Abdominal/pelvic cavity/omentum	27 (18.5)
Others*	22 (15.1)
Long diameter of tumor (cm)	
≤ 2	12 (8.2)
2–5	37 (25.3)
5–10	63 (43.2)
> 10	34 (23.3)
Mitosis	
≤ 5/50HPF	66 (45.2)
6–10/50HPF	50 (34.2)
> 10/50HPF	30 (20.5)
CD117 expression	
Positive	108 (74.0)
Negative	31 (21.2)
NA	7 (4.8)
DOG-1 expression	
Positive	66 (45.2)
Negative	27 (18.5)
NA	53 (36.3)
CD34 expression	
Positive	96 (65.8)
Negative	36 (24.6)
NA	14 (9.6)

Note: *including colon, rectum, renal, etc. NA: none available.

### Quality control of next-generation sequencing

Average coverage of next-generation sequencing was > 200× and sequence content of four bases T, C, A, G was well called and balanced. The actual GC distribution over all sequences was similar with theoretical distributions. Furthermore, the proportion of N appearing in a sequence was low and the distribution of fragment sizes was uniform (chiefly 100 bp; range: 99–101 bp). This guaranteed accuracy of sequencing and established a foundation for data elucidation (Supplementary Figure S2).

### Variants of 48 genes in 146 KIT/PDGFRA wild-type GISTs

Among the 146 patients, 119 had at least one nonsynonymous or deletion variant in the captured gene set with a median variant of 2 (range: 0–34; Supplementary Figure S3). For 48 captured genes, TP53 (43.15%), ROS1 (20.55%), NF1 (19.86%), ATRX (19.86%), and KIT (19.18%) were the five most frequently variants. Moreover, other variants with prevalence > 10% were identified including BRCA2 (17.12%), BRAF (16.44%), TSC1 (15.07%), MET (15.07%), F5 (13.01%), DEPDC5 (13.01%), PDGFRA (12.33%), SLTM (11.64%), KDR (11.64%), ALK (11.64%), and DDR2 (10.27%) (Supplementary Figure S4). Whether these variants participated in tumorigenesis of GISTs is unclear.

### Variation profile of 48 genes based on clinicopathological features

The heat maps of 48 genes based on different features were analyzed using R software (Supplementary Figure S5–S13). Data from hierarchical cluster analysis for all patients (Figure S5) suggested the following stratified cluster analysis based on sex (Figure S6), age (Figure S7), tumor sites (Figure S8), tumor sizes (Figure S9), mitosis (Figure S10), CD117 (Figure S11), DOG-1 (Figure S12), and CD34 (Figure S13) expression. No obvious differences in variation profile were noted among different features.

### KIT/PDGFRA mutations in KIT/PDGFRA wild-type GISTs and correlations to imatinib sensitivity

For hot spots for *KIT* (exons 9, 11, 13, and 17) and *PDGFRA* (exons 12, and 18) genes, 19 (13.0%) and 4 (2.7%) of 146 KIT/PDGFRA wild-type GISTs patients carried KIT and PDGFRA mutations, respectively, with a mutation ratio (mutratio, mutcount/coverage) less than 25%. The mutation types contained W557G (*n* = 1), W557R (*n* = 2), V559D (*n* = 1), Del 557–558 (*n* = 3), L576P (*n* = 6), Del 579 (*n* = 1) in exon 11 of *KIT* gene, A814S (*n* = 1), N822K (*n* = 4) in exon 17 of *KIT* gene, and R585K (*n* = 1) in exon 12 of *PDGFRA* gene, D842V (*n* = 2), D842Y (*n* = 1) in exon 18 of *PDGFRA* gene (Table 2). These mutations were mutually exclusive, and all mutation types in exon 11, N822K in exon 17 of the *KIT* gene, and D842V in exon 18 of the *PDGFRA* gene were frequently reported in GISTs. Based on our previous large scale analysis [13], only one mutation type codon (a 502–503 duplication) was found in exon 9 of the *KIT* gene. No mutation in exon 9 was found in this study and mutations were not found in exon 13 of the *KIT* gene.

**Table 2:** Hot spots mutations found by next-generation sequencing in KIT/PDGFRA wild-type GISTs

Case	Exon of gene	Mutation type	MutRatio*
2014-BZ0157	11 of KIT	Del 557–558	12.1%
2014-BZ0027	11 of KIT	L576P	20.4%
2014-BZ0129	11 of KIT	L576P	17.7%
2014-BZ0132	11 of KIT	W557R	22.8%
2014-BZ0069	11 of KIT	Del 557–558	16.9%
2014-BZ0184	11 of KIT	L576P	11.8%
2014-BZ0020	11 of KIT	W557R	24.1%
2014-BZ0075	11 of KIT	Del 579	14.4%
2014-BZ0093	11 of KIT	W557G	18.4%
2014-BZ0019	11 of KIT	V559D	23.5%
2014-BZ0021	11 of KIT	L576P	10.5%
2014-BZ0128	11 of KIT	L576P	11.7%
2014-BZ0166	11 of KIT	L576P	13.4%
2014-BZ0017	11 of KIT	Del 557–558	11.0%
2014-BZ0028	17 of KIT	N822K	11.9%
2014-BZ0162	17 of KIT	N822K	19.8%
2014-BZ0096	17 of KIT	A814S	10.8%
2014-BZ0024	17 of KIT	N822K	22.8%
2014-BZ0135	17 of KIT	N822K	10.1%
2014-BZ0127	12 of PDGFRA	R585K	22.9%
2014-BZ0015	18 of PDGFRA	D842V	19.1%
2014-BZ0114	18 of PDGFRA	D842Y	10.3%
2014-BZ0038	18 of PDGFRA	D842V	18.1%

Note:

* MutRatio = MutCount/Coverage ×100%.

Among 146 patients, 12 received imatinib palliative therapy and 11 patients (91.7%) were evaluable for clinical response. Two of 3 patients (66.7%) with KIT mutations identified by NGS achieved partial response (PR), while only 1 of 8 patients (12.5%) without KIT mutations reached PR, suggesting NGS could identify a portion of patients eligible for imatinib therapy.

**Figure 1:**
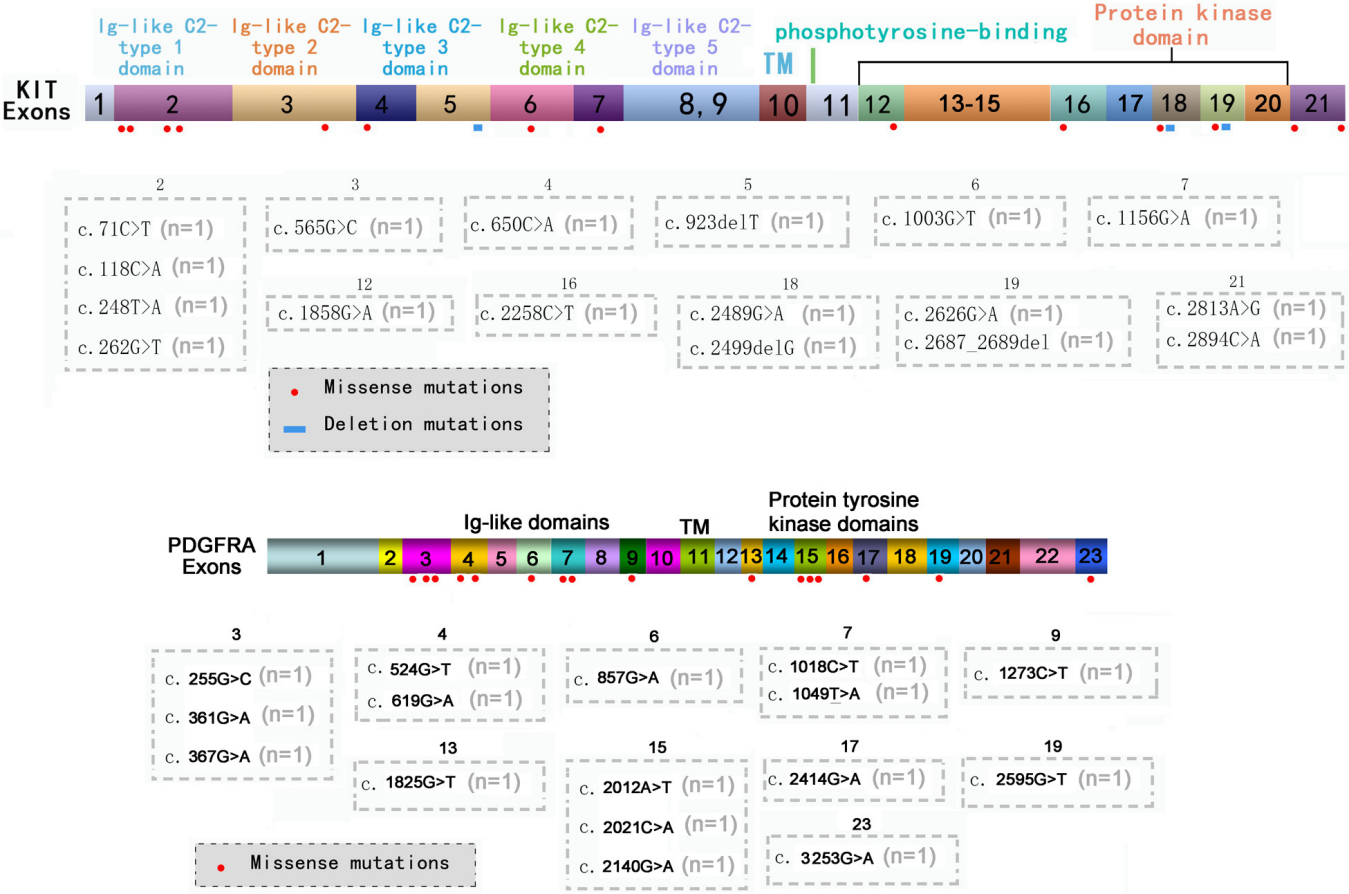
Mutations located in other exons of KIT/PDGFRA genes The distribution of missense or deletion mutations identified by NGS in other exons of *KI*T (**A**) or *PDGFRA* (**B**) genes.

In addition to hot spots, other exons of the *KIT* or *PDGFRA* genes were confirmed to carry missense or deletion mutations (Figure 1), which could co-exist with each other and with mutations in hot spots. A total of 19 patients were confirmed to carry 33 types of mutations in other exons of the *KIT* or *PDGFRA* genes. Six patients carried mutations both in hot spots and in other exons of the *KIT* or *PDGFRA* genes but the potential functions of these mutations are unclear.

### Intratumoral KIT mutational heterogeneity

**Figure 2:**
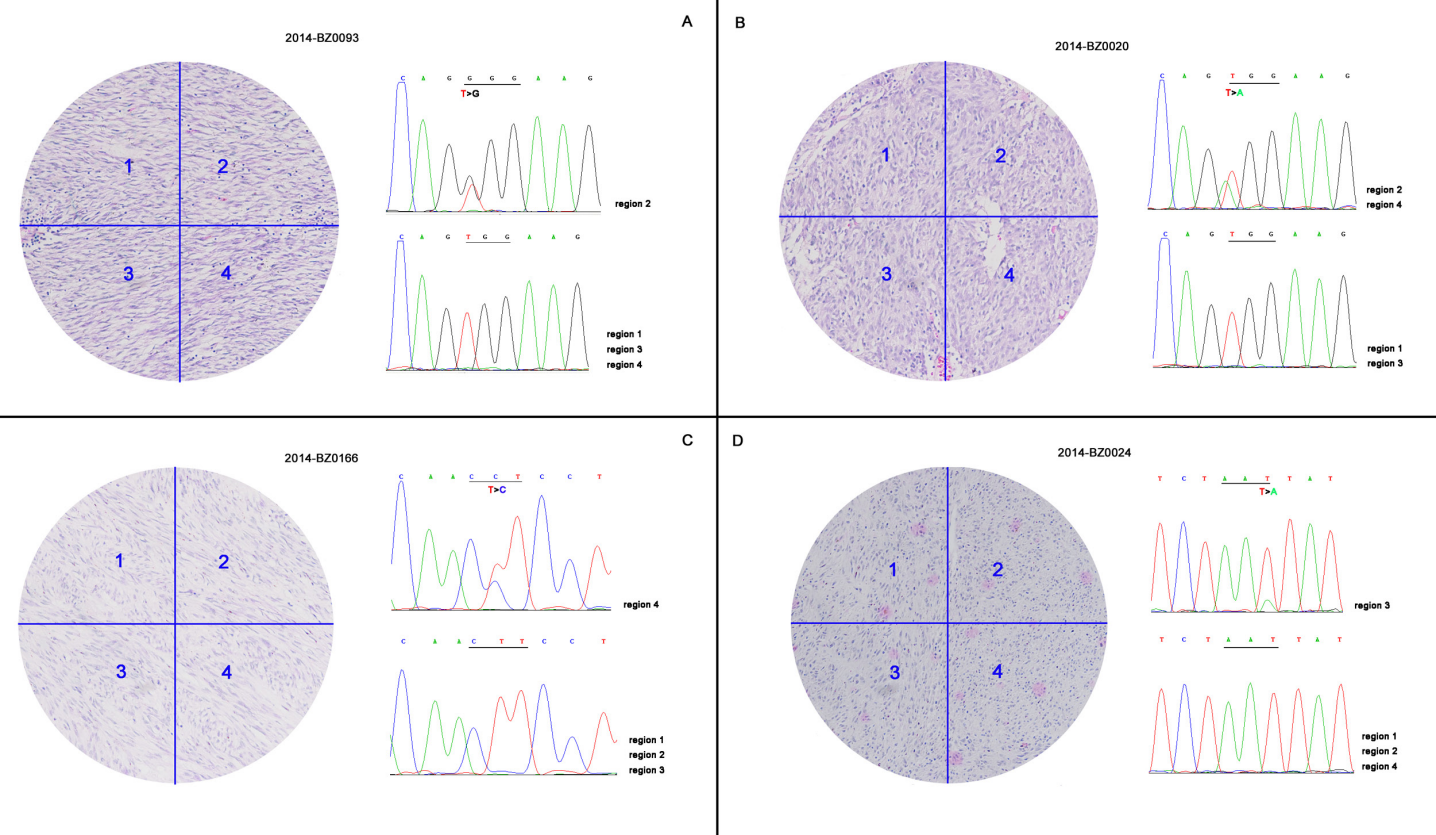
Intratumoral KIT mutational heterogeneity of 4 patients FFPE sections of 19 patients identified to carry hot spots mutations of KIT by NGS were macrodissected into four regions followed by PCR amplification and Sanger sequencing. Four of 19 patients demonstrated intratumoral KIT mutational heterogeneity with concurrent wild-type and mutant tumor cells.

Data show that 19 KIT/PDGFRA wild-type patients carried mutations in hot spots of the *KIT* gene, which were not identified using Sanger sequencing. Polyclonal features of KIT mutations have been reported previously, and consequently, formalin-fixed paraffin-embedded sections of each patient were macrodissected into four regions based on H & E staining. Genomic DNA was extracted from macrodissected samples followed by PCR amplification and Sanger sequencing. Data show that 4 of 19 patients had intratumoral KIT mutational heterogeneity (Figure 2). In addition to wild-type cells, four patients carried mutant cells with different mutation types containing W557G, W557R, L576P, and N822K, data consisted with Table 2 data. The mutational heterogeneity potentially triggered mechanisms of polyclonal evolution and metastasis, as well as different therapeutic sensitivities.

### KRAS and BRAF mutations in KIT/PDGFRA wild-type GISTs

Important downstream molecules of KIT/PDGFRA, KRAS and BRAF mutations have been reported in different studies. For common mutations of KRAS (exon 2) and BRAF (exon 15), one patient carried a G13D mutation in exon 2 of the *KRAS* gene, and six carried mutations (5 carried V600E mutations and one carried the A598D mutation) in exon 15 of the *BRAF* gene in this study, and this excluded hot spots of *KIT/PDGFRA* genes. Various mutations were also observed in other exons of *KRAS* and *BRAF* genes, which could co-exist with each other and with common mutations (Supplementary Table S2).

## DISCUSSION

Oncogenic mutations of *KIT*/*PDGFRA* genes are the major mechanisms of tumorigenesis of GISTs, and *KIT/PDGFRA* genotypes are correlated to imatinib efficacy [3]. At present, patients with KIT/PDGFRA wild-type GISTs are usually not treated with imatinib but 30% of these patients may benefit from imatinib, likely due to unidentified susceptibility factors or incomplete sequencing data with Sanger sequencing. Here, we sequenced whole exomes of 48 genes containing KIT/PDGFRA with targeted NGS in a sample of KIT/PDGFRA wild-type GISTs.

For hot spots in the *KIT* and *PDGFRA* genes, 19 KIT mutations and 4 PDGFRA mutations were identified in KIT/PDGFRA wild-type cases according NGS. These mutations were mutually exclusive and most mutations (*n* = 20) were point mutations. Mutations in exons 9 and 13 of the *KIT* gene were not identified. NGS may not be suitable for identifying deletion mutations in large fragments located in exon 11 of the *KIT* gene. Xu and colleagues reported that *KIT* and *PDGFRA* mutations in 121 samples by NGS were 49.6% and 0.8%, respectively [14], significantly fewer mutations than previously reported [7, 13]. However, Gleeson FC and colleagues reported that targeted NGS of cytology samples from 19 patients with GISTs is clinically feasible [15]. Our data show that NGS did not identify deletion mutations in exon 11 of the *KIT* gene in several samples. Thus, NGS should be optimized for clinical practice for assessing GISTs.

The five most frequently mutated genes were TP53, ROS1, NF1, ATRX, and KIT, but whether these mutation genes participate in tumorigenesis of GISTs warrants more study. Hechtman's group analyzed 8 patients with wild-type KIT/PDGFRA, and 8 cases had loss of SDHB expression and carried ARIDIA, TP53, and other gene alterations [16]. Pantaleo and colleagues reported that SDH mutations were frequently observed in patients with KIT/PDGFRA wild-type GISTs [5], especially in pediatric patients [4]. Patients with wild-type KIT/PDGFRA had high expression of IGF pathway family members [17, 18], which offered an alternative therapeutic strategy for treating KIT/PDGFRA wild-type GISTs. Studies with small samples indicated that genomic profiles between KIT/PDGFRA wild-type and mutant GISTs were different [19, 20]. Until now, a pathogenic mechanism to explain KIT/PDGFRA wild-type GISTs was not known and comparative analyses of whole genomic sequencing of large samples of KIT/PDGFRA wild-type and mutant GISTs should provide insights about KIT/PDGFRA wild-type GISTs.

Hot spots in *KIT/PDGFRA* genes were mutually exclusive and mutations in other exons of the *KIT/PDGFRA* gene co-existed with each other and with hot spot mutations. Mechanisms or reasons for exclusive hot spot mutations were similar to KRAS/BRAF exclusive mutations in colorectal cancer [21]. For GISTs, only primary hot spot mutations in *KIT/PDGFRA* genes were excluded, however, primary and secondary mutations were concomitantly located in hot spots of the *KIT/PDGFRA* genes [22].

Previous research suggests that KIT mutations have polyclonal features [2, 23], so FFPE sections of 19 patients identified to carry hot spots mutations of the *KIT* gene were macrodissected into four regions followed by PCR amplification and Sanger sequencing. Four of the 19 cases had intratumoral *KIT* mutational heterogeneity with concurrent wild-type and mutant tumor cells, which may have triggered polyclonal evolution and metastasis and unique therapeutic sensitivity.

Downstream pathways of *KIT/PDGFRA* genes may be important sources for explaining drug sensitivity. KRAS and BRAF mutations have been reported but data were inconsistent [8, 24]. Miranda C and colleagues reported that KRAS (5%) and BRAF (2%) mutations were identified in GISTs carrying KIT/PDGFRA mutations, not in KIT/PDGFRA wild-type GISTs [8]. Agaimy A and colleagues demonstrated BRAF mutations (7%) were detected in KIT/PDGFRA wild-type GISTs not in mutant GISTs [25]. Our previous results presented that KRAS (1.7%) and BRAF (1.7%) mutations were detected in KIT/PDGFRA wild-type GISTs not in mutant GISTs (unpublished data). Whether KRAS/BRAF mutations can predict imatinib resistance in GISTs requires validation in a larger sample size.

Nannini and coworkers described KIT/PDGFRA wild-type GIST as a set of different diseases sustained by specific molecular alterations not yet known [25]. Although our results were somewhat superficial, we identified patients eligible for imatinib therapy by NGS. Intratumoral *KIT* mutational heterogeneity may be monitored to evaluate imatinib efficacy.

## MATERIALS AND METHODS

### Patients and samples

**Figure 3:**
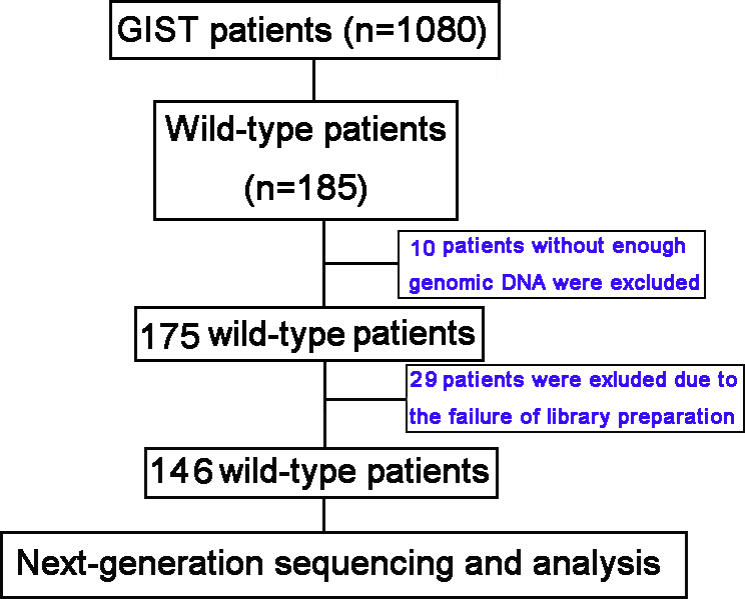
Patient screening flow chart From 1,080 patients studied for KIT/PDGFRA mutations, 185 were KIT/PDGFRA wild-type and 146 were analyzed using targeted next-generation sequencing (NGS). There was insufficient genomic DNA or the library preparation failed for 39 patients.

From October 2001 to January 2014, a total of 1,080 individuals with GISTs were screened for KIT or PDGFRA mutations at Peking University Cancer Hospital. We identified 185 KIT/PDGFRA wild-type patients and among these 39 were excluded due to lack of genomic DNA data or failure of library preparation for NGS (Figure 3). All clinicopathological features and treatments were retrospectively assessed from medical records, and samples were taken prior to imatinib or sunitinib treatment. Written informed consent was obtained from all patients for sample study, and the study was approved by the Medical Ethics Committee of Peking University Cancer Hospital.

### DNA extraction and mutation detection of *KIT* and *PDGFRA* genes

Genomic DNA was extracted from formalin-mixed paraffin-embedded tumor specimens with tumor cells > 50% based on H & E staining using QIAamp DNA FFPE Tissue Kit (Qiagen, Hilden, Germany), and stored at −80°C for future use after quantification with Nanodrop 2000. Mutational analysis in exons 9, 11, 13, and 17 of *KIT* gene and exons 12, and 18 of *PDGFRA* gene was performed with PCR amplification and Sanger sequencing according to published procedures [7]. Each sample was sequenced at least twice.

### Library preparation and targeted NGS

Genomic DNA (3 μg) was used for library preparation according to the manufacturer's instruction (MyGenostics, Beijing, China), and the final library size of 350–450 bp, containing adapter sequences was used in the following experiment.

A panel of 48 genes (Supplementary Table S1) including *KIT* and *PDGFRA* genes was captured with OncoCap Enrichment System (MyGenostics, Beijing, China) based on previously published methods [26]. After enrichment, libraries were sequenced on an Illumina Solexa HiSeq 2000 sequencer for paired reads of 100 bp followed by data retrieval using Solexa QA package and a cutadapt program (http://code.google.com/p/cutadapt/).

### Bioinformatic analysis

Supplementary Figure S1 shows the experimental design. Briefly, illumina clean reads (the sequencing quality > 20 and read length > 80 bp) were aligned to each human reference genome (hg19) using the BWA program and quality scores were recalibrated and realigned to references using GATK software. Duplicated reads were removed using Sequence Alignment/Map tools (SAMtools) and only uniquely mapping reads were used for variation assessment. Low frequency variants were identified on the basis of the bam file. The SAMtools mpileup command was used to generate pileup files. VarScan was performed to assess pileup files from tumor samples to heuristically call for a genotype at positions achieving certain thresholds of coverage and quality.

SNVs were detected and genotyped with the GATK UnifiedGenotyper in single-sample mode, and variants were filtered with GATK VariantFiltration module (with filters “QUAL < 50.0 & QD < 5.0 & HRun > 10 & DP < 4” and parameters –cluster 3 -window 10). Indels were detected with GATK IndelGenotyperV2 and filtered with a custom python module that removed sites with amax_cons_av ≥ 1.9 (maximum average number of mismatches across reads supporting the indel) or max_cons_nqs_av_mm ≥ 0.2 (maximum average mismatch rate in the 5-bp NQS window around the indel, across indel-supporting reads).

Low frequency variants were identified with Fisher's exact test. Post-calling filters are based on read depth, sequencing quality, mismatches, and overlap with indels. Variation annotations such as locations (exonic, intronic and intergenic region) and effects on protein coding (synonymous, missense, nonsense, frameshift), were performed with an in-house developed bioinformatics tool with RefSeq (hg19, from UCSC) and UCSC annotation (http://www.ncbi.nlm.nih.gov/refseq/). Variants with the following conditions could be analyzed: (1) located within an exonic or splicing region; (2) nonsynonymous; (3) MAF < 0.05 in the European 1,000 genomes variant database; (4) reads supporting the variation ≥ 5; and (5) variation frequency > 0.01.

### Experimental validation

Macrodissection of tumor sections and subsequent PCR amplification and Sanger sequencing was used to validate detected missense mutations by NGS. PCR products were sequenced with a 3730XL genetic analyzer and Chromas software was used to analyze sequencing results.

## SUPPLEMENTARY MATERIALS TABLES AND FIGURES




