# Supplementary material for: Intratumoral KIT mutational heterogeneity and recurrent KIT/ PDGFRA mutations in KIT/PDGFRA wild-type gastrointestinal stromal tumors
Source: Oncotarget. 2016 Feb 2;7(21):30241–9. doi: 10.18632/oncotarget.7148 (PMC5058677; doi:10.18632/oncotarget.7148)
Supplement: Supplementary file 1 [file oncotarget-07-30241-s001.pdf]

Intratumoral KIT mutational heterogeneity and recurrent KIT/PDGFRA mutations in KIT/PDGFRA wild-type gastrointestinal stromal tumors

Supplementary Materials

Supplementary Table S1: The list of 48 genes

AKT1	DDR2	KIT	RET
AKT2	DEPDC5	KRAS	ROS1
ALK	DPYD	LKB1	TERT
ATRX	EGFR	MEK1	TP53
BIM	F2	MEK2	TPMT
BRAF	F5	MET	TSC1
BRCA1	FGFR1	NF1	TSC2
BRCA2	FUBP1	NPRL2	UGT1A1
CDKN2A	HER2	NRAS	VEGFA
CDKN2B	HRAS	PDGFRA	VEGFR1
CIC	IDH1	PIK3CA	VEGFR2
DAXX	IDH2	PTEN	VEGFR3

Supplementary Table S2: KRAS and BRAF variations identified in this study by next-generation sequencing

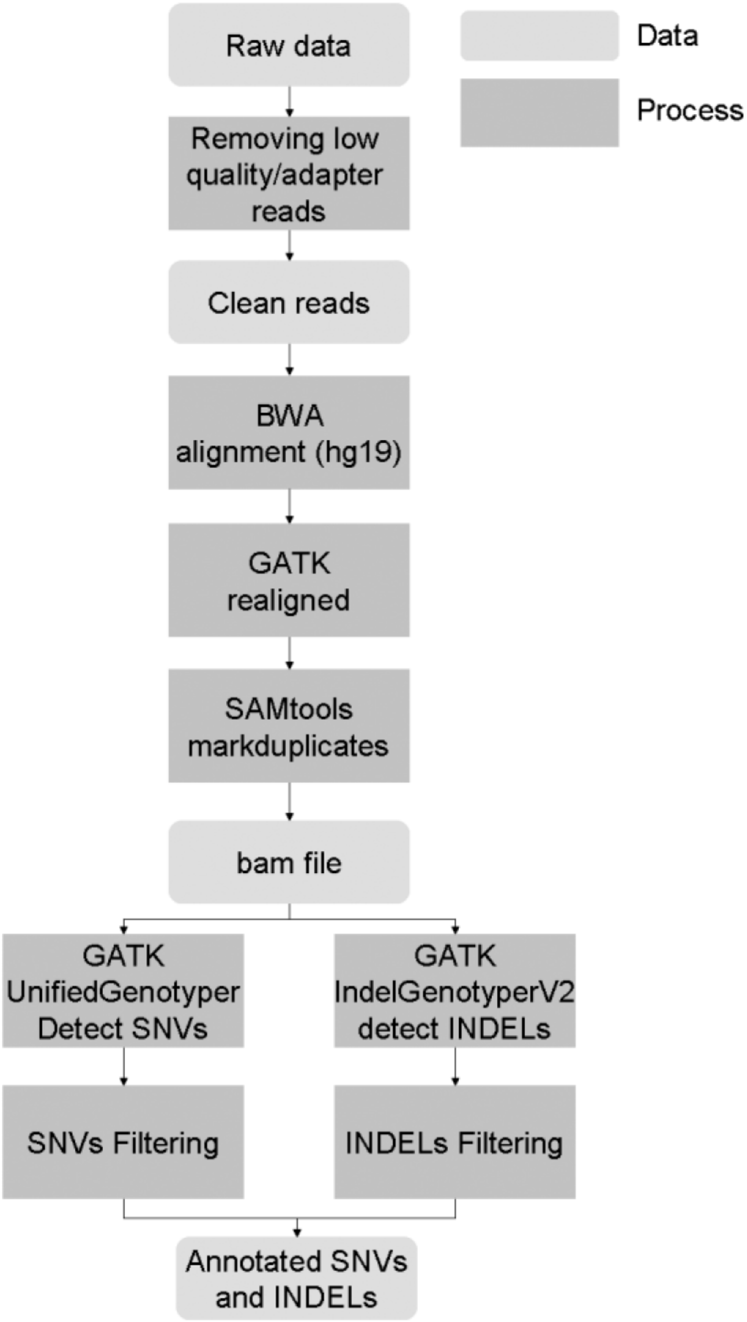

Supplementary Figure S1: The work flow of gene variation analysis.

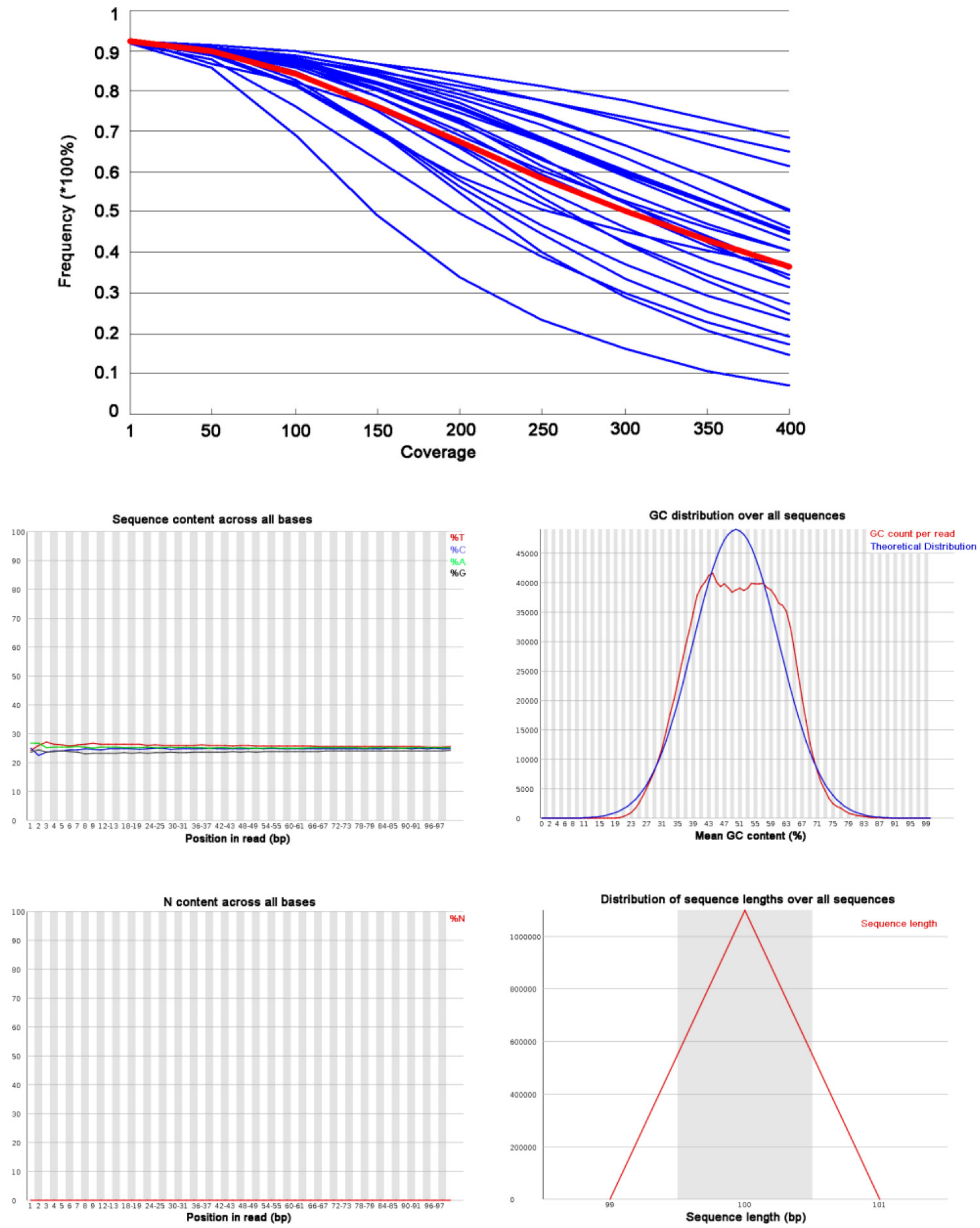

Supplementary Figure S2: Quality control of next-generation sequencing. All parameters (including average coverage of sequencing, sequence content of four bases, actual GC distribution, the proportion of N appearing, and the distribution of fragment sizes) guaranteed the accuracy of sequencing and established the foundation for data elucidation.

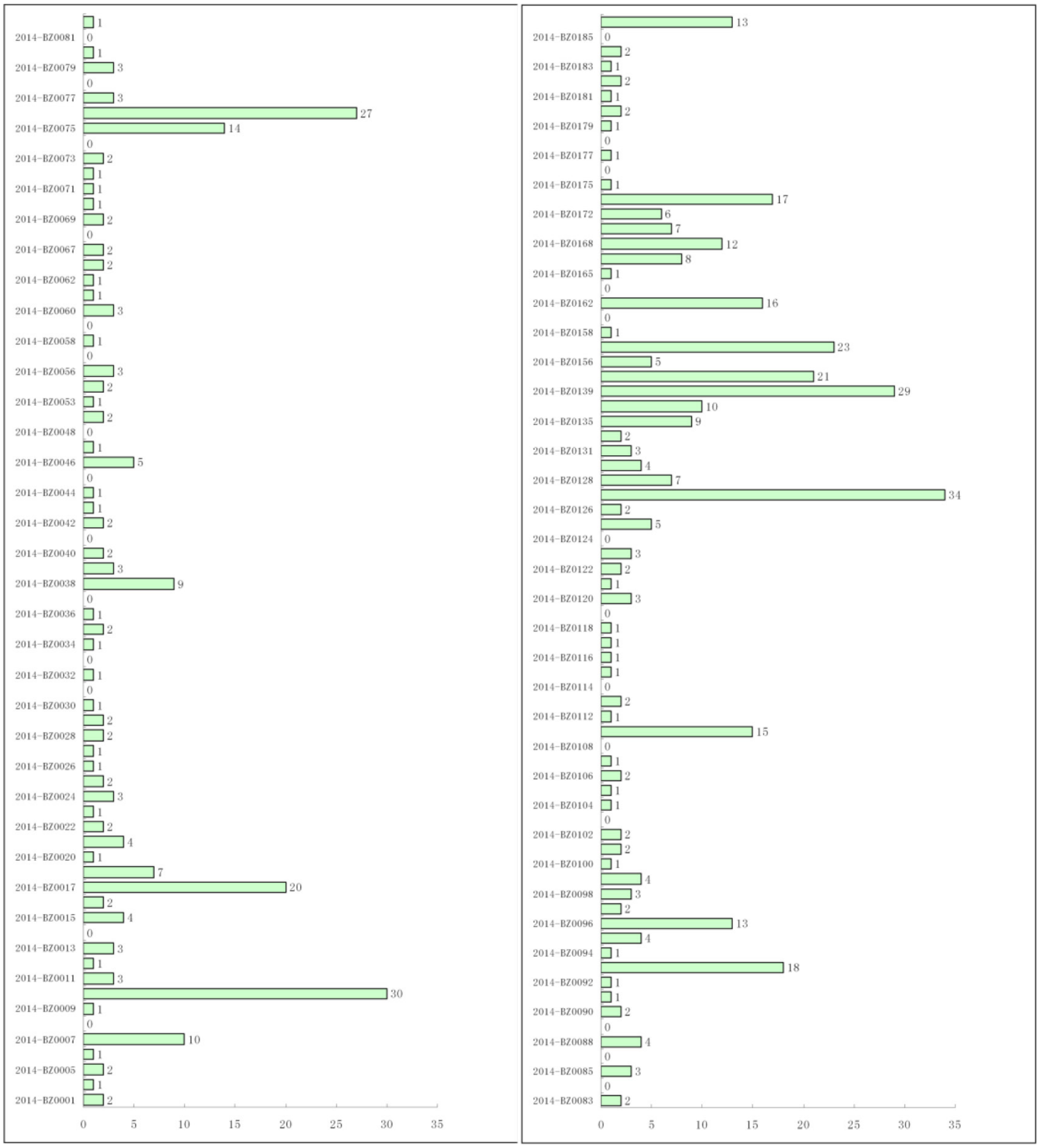

Supplementary Figure S3: The number of variants in 146 patients. Among all patients, 119 cases had at least one nonsynonymous or deletion variant in the captured gene set. The number of variants in all patients ranged from 0 to 34 with a median of 2.

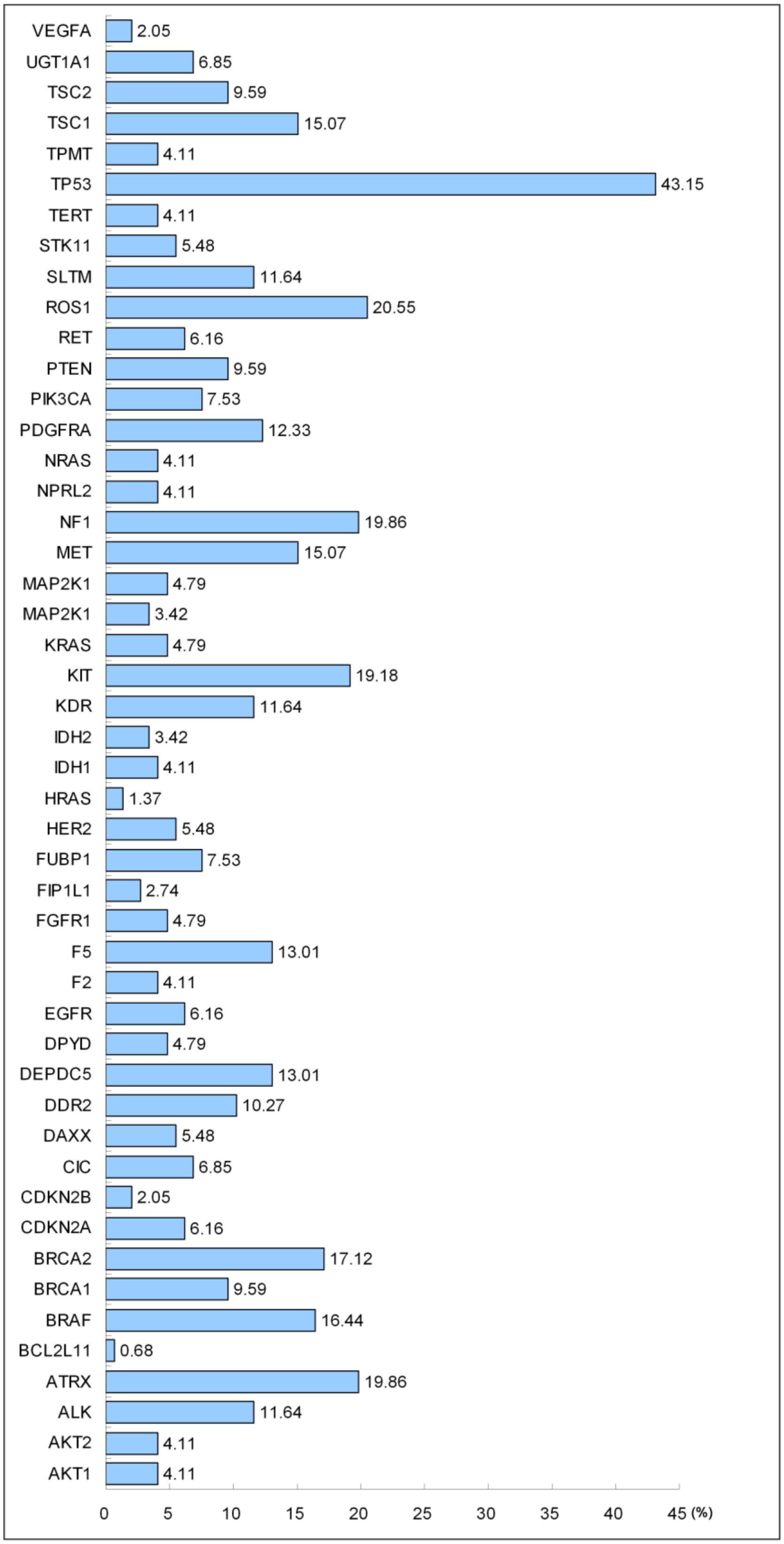

Supplementary Figure S4: The frequency of variants in our cohort. Whether these variants participated in tumorigenesis of GISTs need to be further investigated.

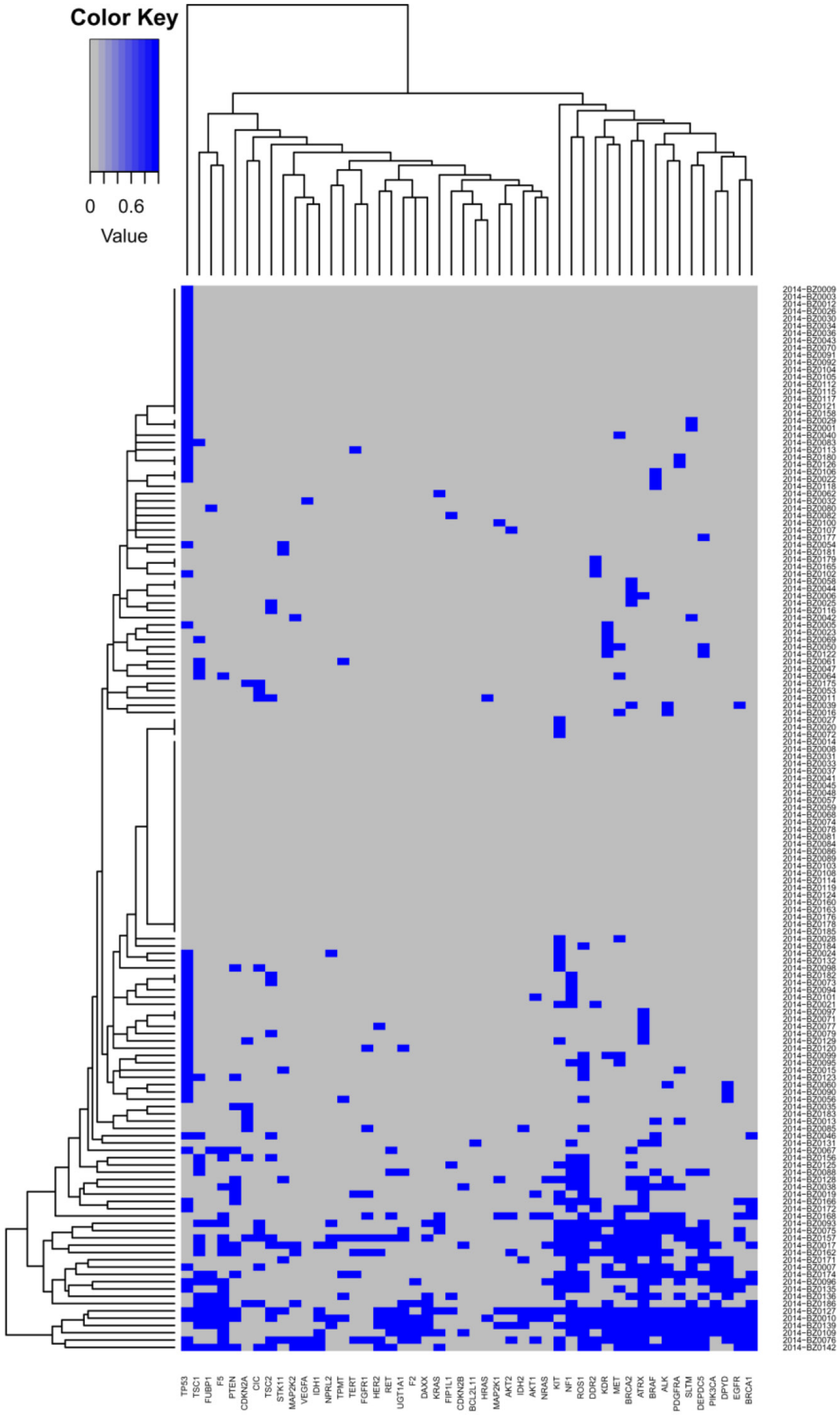

Color Key
0
0.6
Value
TP53
TSC1
FUBP1
F5
PTEN
CDKN2A
CIC
TSC2
STK11
MAP2K2
VEGFA
IDH1
NPRL2
TPMT
TERT
FGFR1
HER2
RET
UGT1A1
F2
DAXX
KRAS
FIP1L1
CDKN2B
BCL2L11
HRAS
MAP2K1
AKT2
IDH2
AKT1
NRAS
KIT
NF1
ROS1
DDR2
KDR
MET
BRCA2
ATRX
BRAF
ALK
PDGFRA
SLTM
DEPDC5
PIK3CA
DPYD
EGFR
BRCA1

8

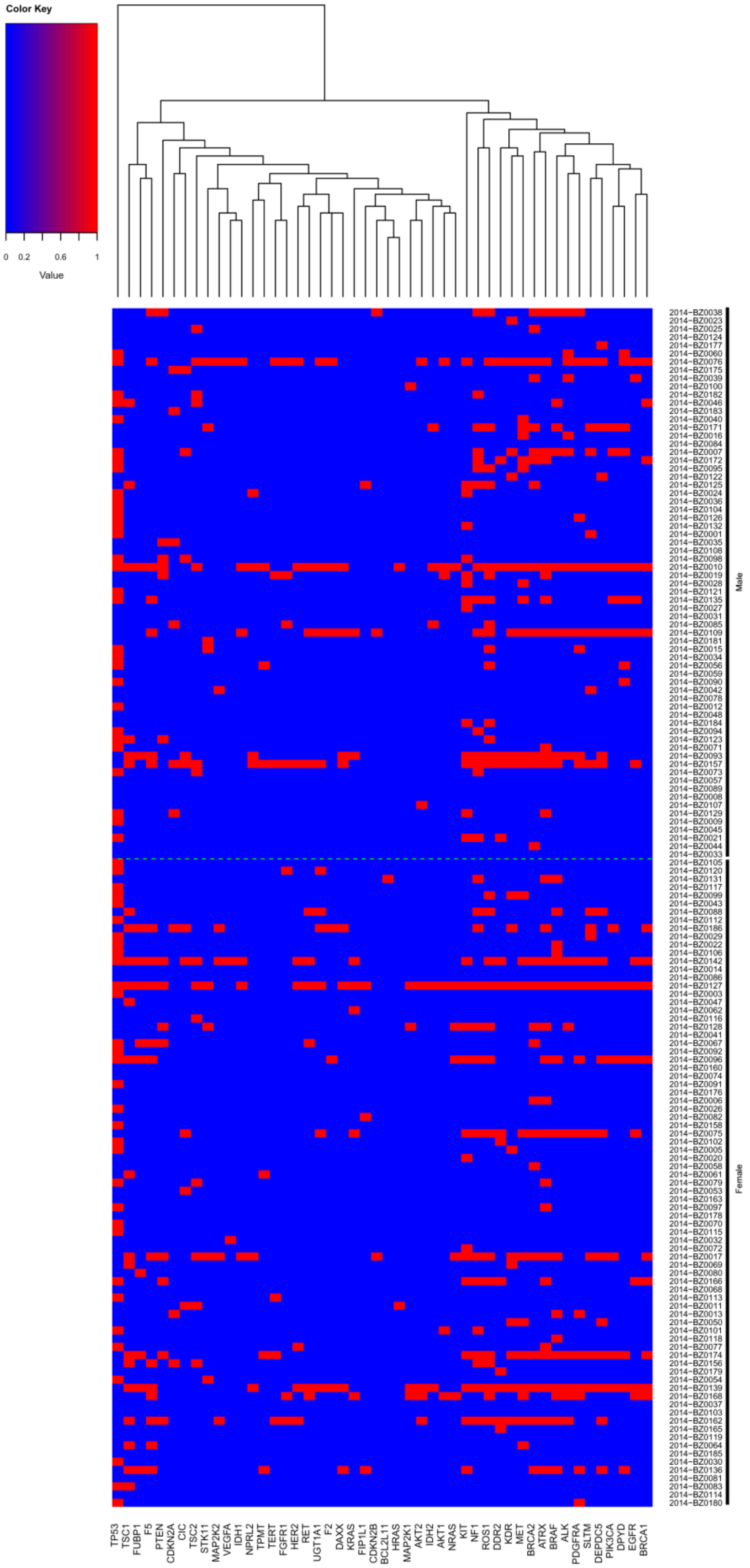
Color Key
0 0.2 0.6 1
Value
Male
Female
TP53
TSC1
FUBP1
F5
PTEN
CDKN2A
CIC
TSC2
STK11
MAP2K2
VEGFA
IDH1
NPRL2
TPMT
TERT
FGFR1
HER2
RET
UGT1A1
F2
DAXX
KRAS
FIP1L1
CDKN2B
BCL2L11
HRAS
MAP2K1
AKT2
IDH2
AKT1
NRAS
KIT
NF1
ROS1
DDR2
KDR
MET
BRCA2
ATRX
BRAF
ALK
PDGFRA
SLTM
DEPDC5
PIK3CA
DPYD
EGFR
BRCA1

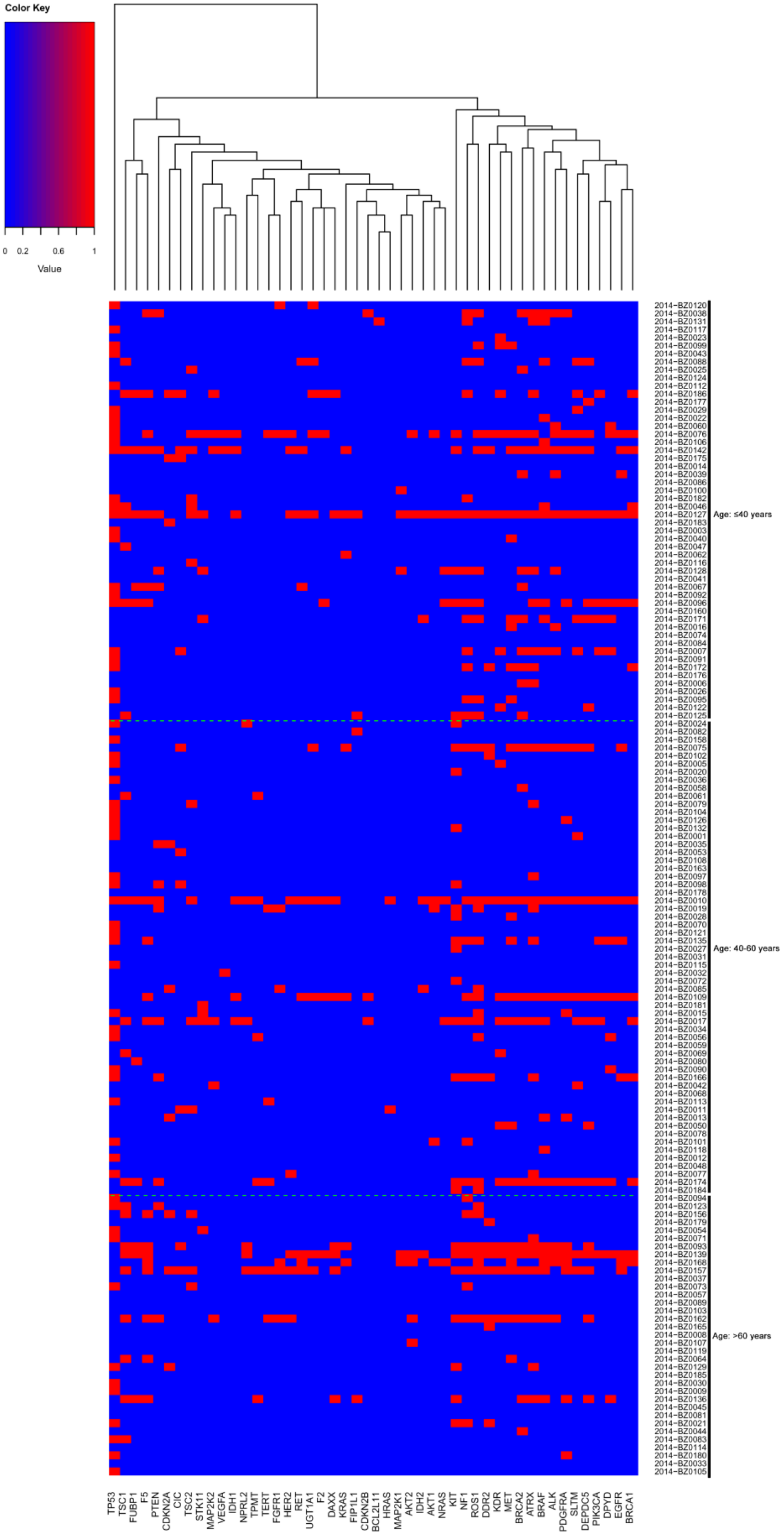
Color Key
0 0.2 0.6 1
Value
Age: ≤40 years
Age: 40-60 years
Age: >60 years
TP53
TSC1
FUBP1
F5
PTEN
CDKN2A
CIC
TSC2
STK11
MAP2K2
VEGFA
IDH1
NPRL2
TPMT
TERT
FGFR1
HER2
RET
UGT1A1
F2
DAXX
KRAS
FIP1L1
CDKN2B
BCL2L11
HRAS
MAP2K1
AKT2
IDH2
AKT1
NRAS
KIT
NF1
ROS1
DDR2
KDR
MET
BRCA2
ATRX
BRAF
ALK
PDGFRA
SLTM
DEPDC5
PIK3CA
DPYD
EGFR
BRCA1

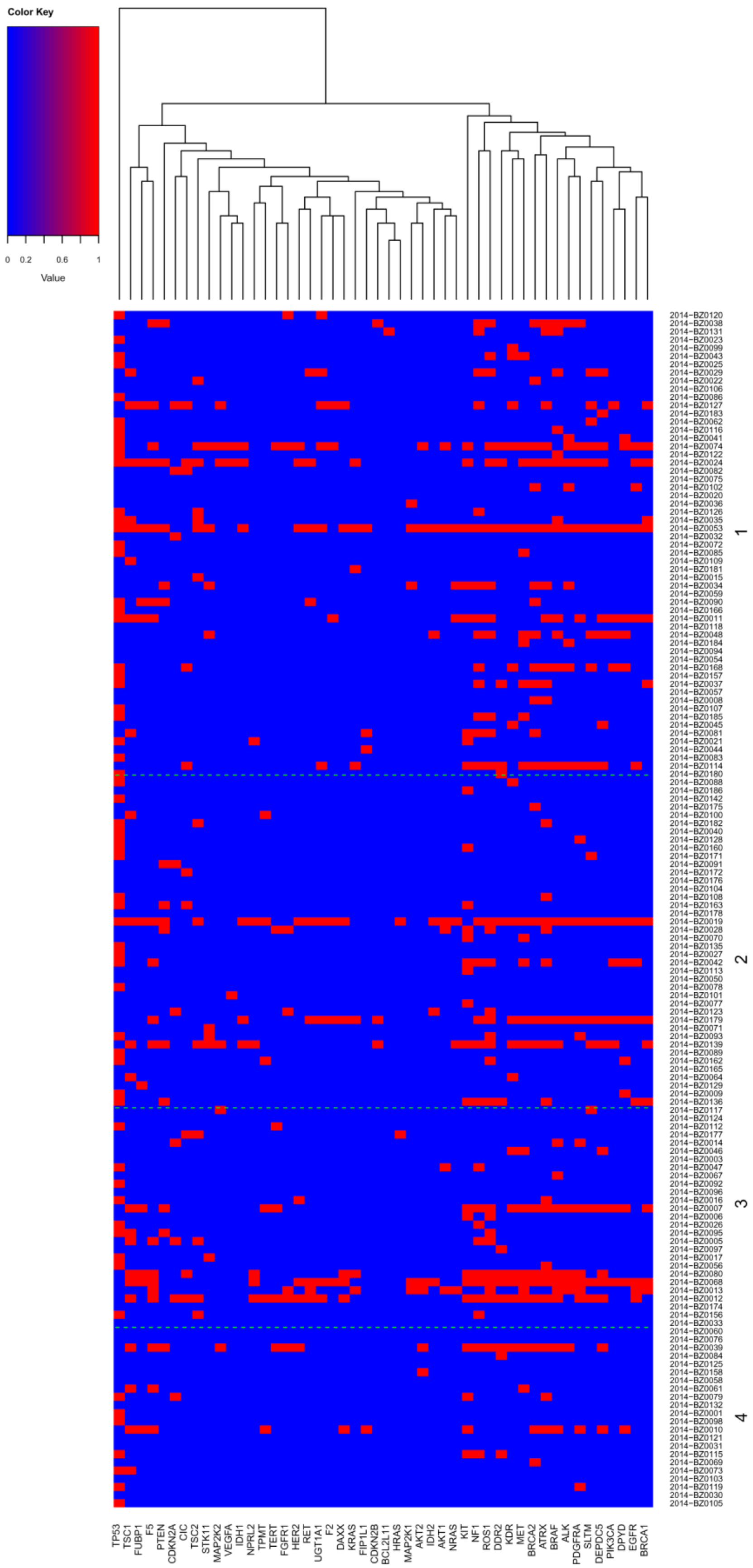

Primary sites: 1, Stomach; 2, Small bowel; 3, Abdominal/pelvic cavity/omentum; 4, Others including colon, rectum, renal, etc.

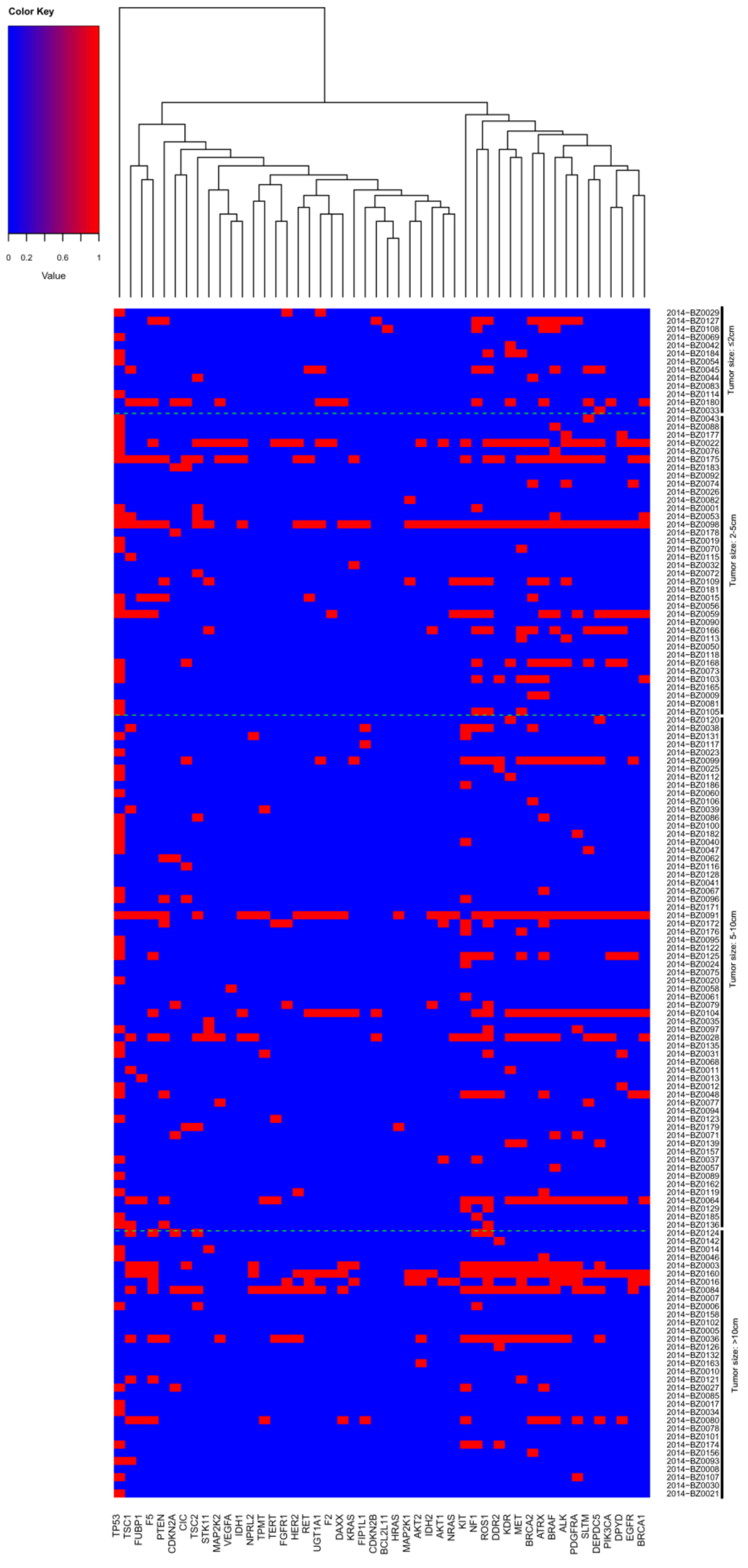

Color Key
0
0.2
0.6
1
Value
2014–BZ0029
2014–BZ0127
2014–BZ0108
2014–BZ0069
2014–BZ0042
2014–BZ0184
2014–BZ0054
2014–BZ0045
2014–BZ0044
2014–BZ0083
2014–BZ0114
2014–BZ0180
2014–BZ0033
Tumor size: ≤2cm
2014–BZ0043
2014–BZ0088
2014–BZ0177
2014–BZ0022
2014–BZ0076
2014–BZ0175
2014–BZ0183
2014–BZ0092
2014–BZ0074
2014–BZ0026
2014–BZ0082
2014–BZ0001
2014–BZ0053
2014–BZ0098
2014–BZ0178
2014–BZ0019
2014–BZ0070
2014–BZ0115
2014–BZ0032
2014–BZ0072
2014–BZ0109
2014–BZ0181
2014–BZ0015
2014–BZ0056
2014–BZ0059
2014–BZ0090
2014–BZ0166
2014–BZ0113
2014–BZ0050
2014–BZ0118
2014–BZ0168
2014–BZ0073
2014–BZ0103
2014–BZ0165
2014–BZ0009
2014–BZ0081
2014–BZ0105
Tumor size: 2-5cm
2014–BZ0120
2014–BZ0038
2014–BZ0131
2014–BZ0117
2014–BZ0023
2014–BZ0099
2014–BZ0025
2014–BZ0112
2014–BZ0186
2014–BZ0060
2014–BZ0106
2014–BZ0039
2014–BZ0086
2014–BZ0100
2014–BZ0182
2014–BZ0040
2014–BZ0047
2014–BZ0062
2014–BZ0116
2014–BZ0128
2014–BZ0041
2014–BZ0067
2014–BZ0096
2014–BZ0171
2014–BZ0091
2014–BZ0172
2014–BZ0176
2014–BZ0095
2014–BZ0122
2014–BZ0125
2014–BZ0024
2014–BZ0075
2014–BZ0020
2014–BZ0058
2014–BZ0061
2014–BZ0079
2014–BZ0104
2014–BZ0035
2014–BZ0097
2014–BZ0028
2014–BZ0135
2014–BZ0031
2014–BZ0068
2014–BZ0011
2014–BZ0013
2014–BZ0012
2014–BZ0048
2014–BZ0077
2014–BZ0094
2014–BZ0123
2014–BZ0179
2014–BZ0071
2014–BZ0139
2014–BZ0157
2014–BZ0037
2014–BZ0057
2014–BZ0089
2014–BZ0162
2014–BZ0119
2014–BZ0064
2014–BZ0129
2014–BZ0185
2014–BZ0136
Tumor size: 5-10cm
2014–BZ0124
2014–BZ0142
2014–BZ0014
2014–BZ0046
2014–BZ0003
2014–BZ0160
2014–BZ0016
2014–BZ0084
2014–BZ0007
2014–BZ0006
2014–BZ0158
2014–BZ0102
2014–BZ0005
2014–BZ0036
2014–BZ0126
2014–BZ0132
2014–BZ0163
2014–BZ0010
2014–BZ0121
2014–BZ0027
2014–BZ0085
2014–BZ0017
2014–BZ0034
2014–BZ0080
2014–BZ0078
2014–BZ0101
2014–BZ0174
2014–BZ0156
2014–BZ0093
2014–BZ0008
2014–BZ0107
2014–BZ0030
2014–BZ0021
Tumor size: >10cm
TP53
TSC1
FUBP1
F5
PTEN
CDKN2A
CIC
TSC2
STK11
MAP2K2
VEGFA
IDH1
NPRL2
TPMT
TERT
FGFR1
HER2
RET
UGT1A1
F2
DAXX
KRAS
FIP1L1
CDKN2B
BCL2L11
HRAS
MAP2K1
AKT2
IDH2
AKT1
NRAS
KIT
NF1
ROS1
DDR2
KDR
MET
BRCA2
ATRX
BRAF
ALK
PDGFRA
SLTM
DEPDC5
PIK3CA
DPYD
EGFR
BRCA1

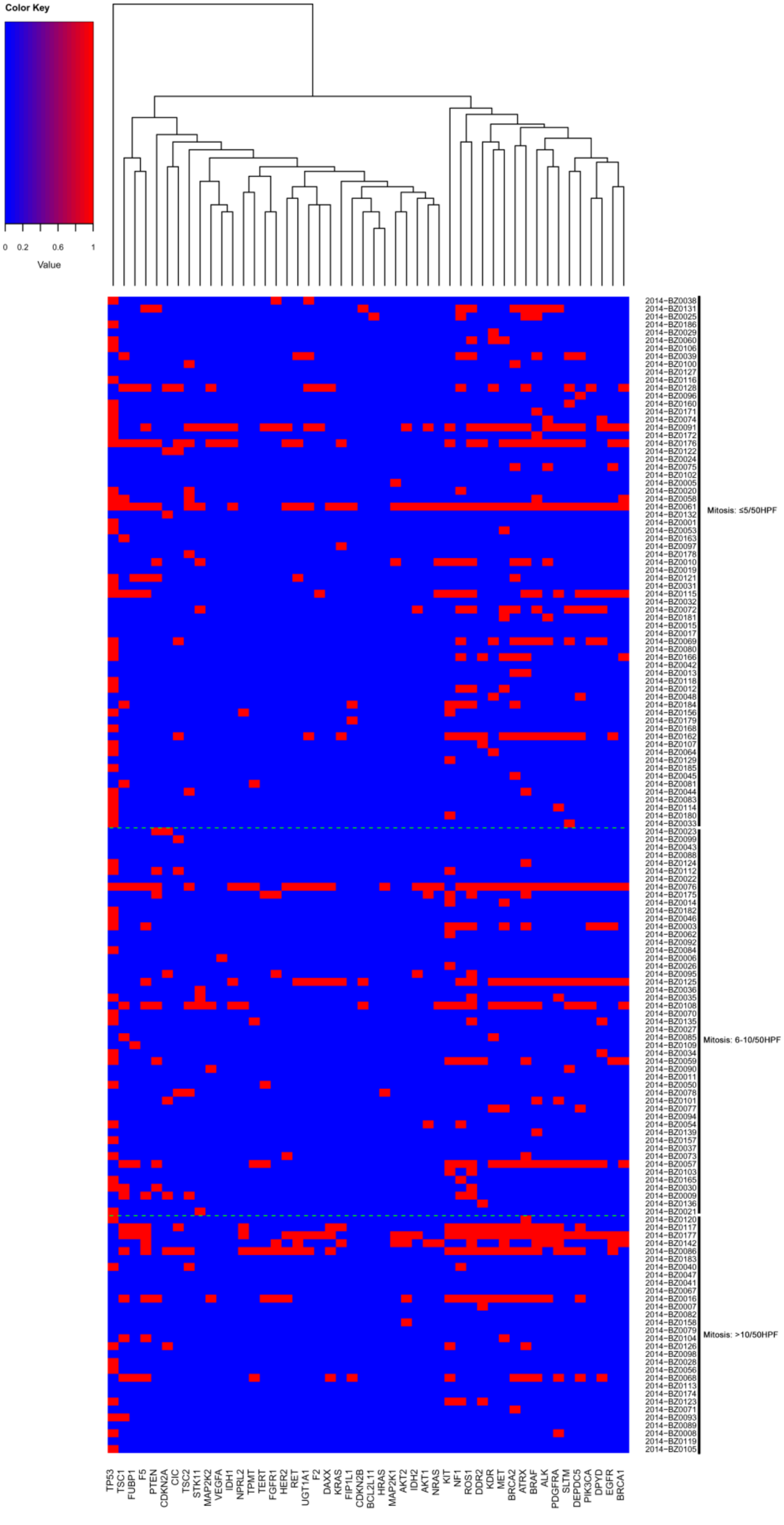
Color Key
0
0.2
0.6
1
Value
Mitosis: ≤5/50HPF
Mitosis: 6-10/50HPF
Mitosis: >10/50HPF
TP53
TSC1
FUBP1
F5
PTEN
CDKN2A
CIC
TSC2
STK11
MAP2K2
VEGFA
IDH1
NPRL2
TPMT
TERT
FGFR1
HER2
RET
UGT1A1
F2
DAXX
KRAS
FIP1L1
CDKN2B
BCL2L11
HRAS
MAP2K1
AKT2
IDH2
AKT1
NRAS
KIT
NF1
ROS1
DDR2
KDR
MET
BRCA2
ATRX
BRAF
ALK
PDGFRA
SLTM
DEPDC5
PIK3CA
DPYD
EGFR
BRCA1

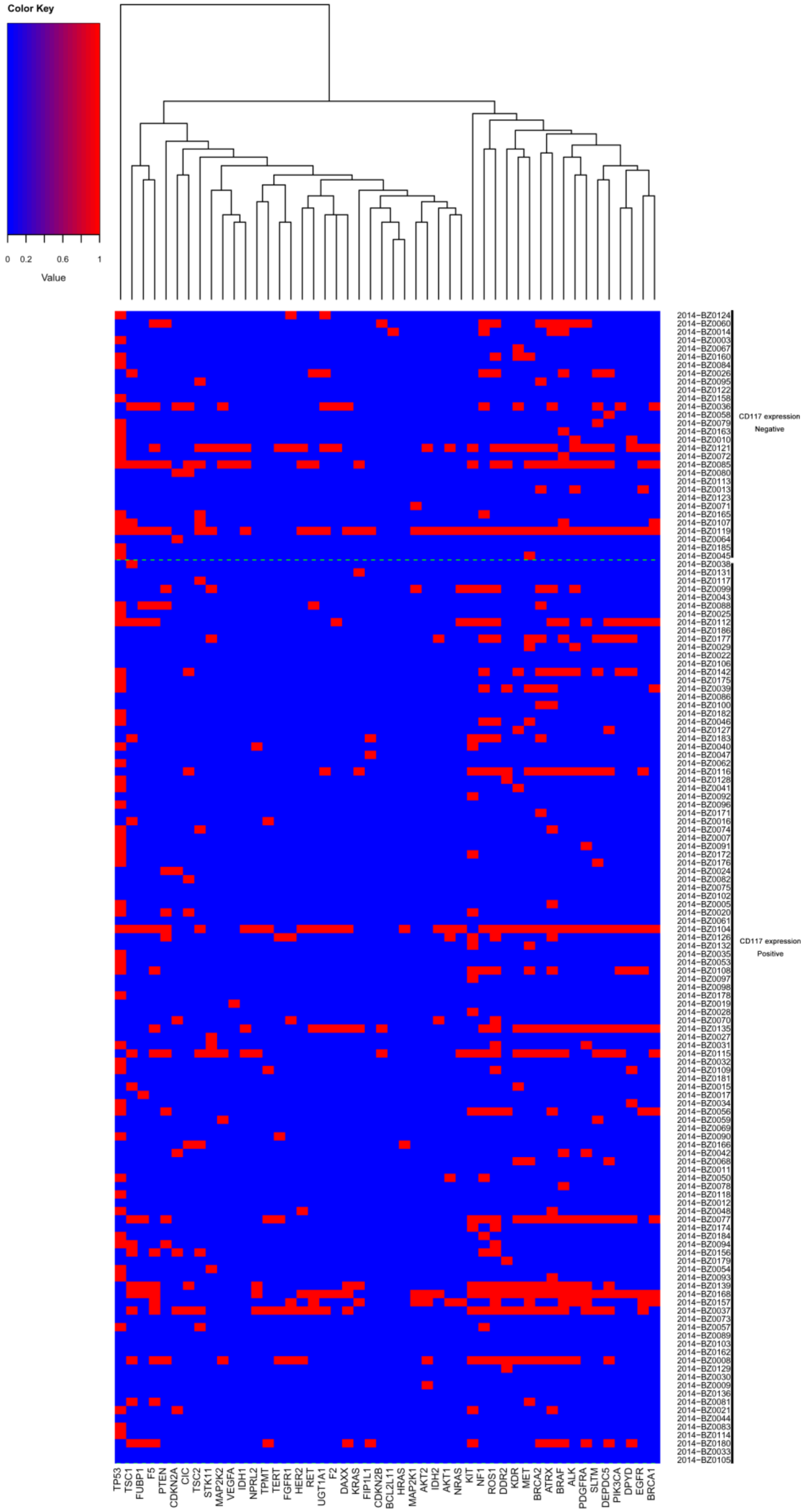

Color Key
0
0.2
0.6
1
Value
CD117 expression
Negative
CD117 expression
Positive
TP53
TSC1
FUBP1
F5
PTEN
CDKN2A
CIC
TSC2
STK11
MAP2K2
VEGFA
IDH1
NPRL2
TPMT
TERT
FGFR1
HER2
RET
UGT1A1
F2
DAXX
KRAS
FIP1L1
CDKN2B
BCL2L11
HRAS
MAP2K1
AKT2
IDH2
AKT1
NRAS
KIT
NF1
ROS1
DDR2
KDR
MET
BRCA2
ATRX
BRAF
ALK
PDGFRA
SLTM
DEPDC5
PIK3CA
DPYD
EGFR
BRCA1

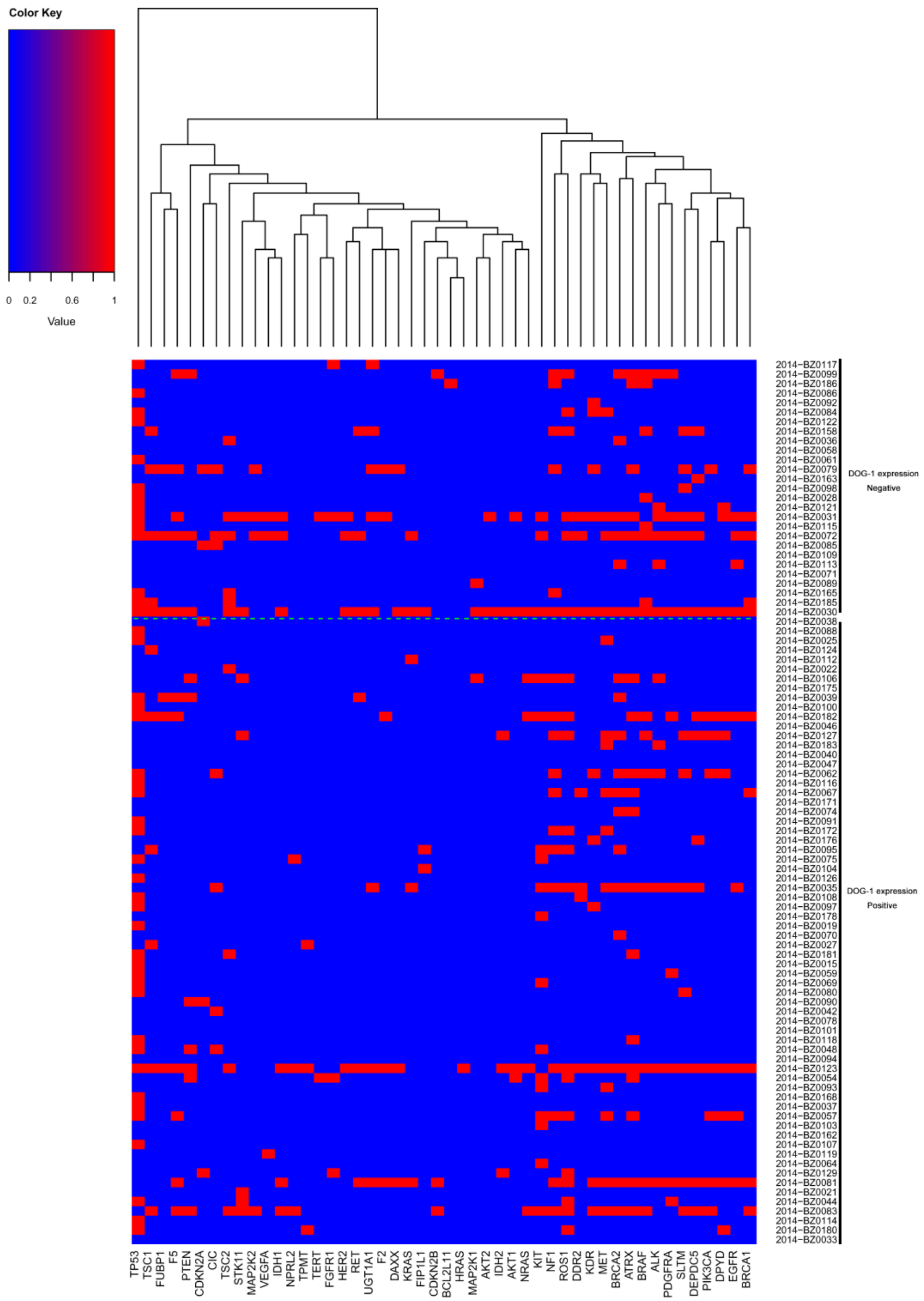

Color Key
0 0.2 0.6 1
Value
2014−BZ0117
2014−BZ0099
2014−BZ0186
2014−BZ0086
2014−BZ0092
2014−BZ0084
2014−BZ0122
2014−BZ0158
2014−BZ0036
2014−BZ0058
2014−BZ0061
2014−BZ0079
2014−BZ0163
2014−BZ0098
2014−BZ0028
2014−BZ0121
2014−BZ0031
2014−BZ0115
2014−BZ0072
2014−BZ0085
2014−BZ0109
2014−BZ0113
2014−BZ0071
2014−BZ0089
2014−BZ0165
2014−BZ0185
2014−BZ0030
2014−BZ0038
2014−BZ0088
2014−BZ0025
2014−BZ0124
2014−BZ0112
2014−BZ0022
2014−BZ0106
2014−BZ0175
2014−BZ0039
2014−BZ0100
2014−BZ0182
2014−BZ0046
2014−BZ0127
2014−BZ0183
2014−BZ0040
2014−BZ0047
2014−BZ0062
2014−BZ0116
2014−BZ0067
2014−BZ0171
2014−BZ0074
2014−BZ0091
2014−BZ0172
2014−BZ0176
2014−BZ0095
2014−BZ0075
2014−BZ0104
2014−BZ0126
2014−BZ0035
2014−BZ0108
2014−BZ0097
2014−BZ0178
2014−BZ0019
2014−BZ0070
2014−BZ0027
2014−BZ0181
2014−BZ0015
2014−BZ0059
2014−BZ0069
2014−BZ0080
2014−BZ0090
2014−BZ0042
2014−BZ0078
2014−BZ0101
2014−BZ0118
2014−BZ0048
2014−BZ0094
2014−BZ0123
2014−BZ0054
2014−BZ0093
2014−BZ0168
2014−BZ0037
2014−BZ0057
2014−BZ0103
2014−BZ0162
2014−BZ0107
2014−BZ0119
2014−BZ0064
2014−BZ0129
2014−BZ0081
2014−BZ0021
2014−BZ0044
2014−BZ0083
2014−BZ0114
2014−BZ0180
2014−BZ0033
DOG-1 expression
Negative
DOG-1 expression
Positive
TP53
TSC1
FUBP1
F5
PTEN
CDKN2A
CIC
TSC2
STK11
MAP2K2
VEGFA
IDH1
NPRL2
TPMT
TERT
FGFR1
HER2
RET
UGT1A1
F2
DAXX
KRAS
FIP1L1
CDKN2B
BCL2L11
HRAS
MAP2K1
AKT2
IDH2
AKT1
NRAS
KIT
NF1
ROS1
DDR2
KDR
MET
BRCA2
ATRX
BRAF
ALK
PDGFRA
SLTM
DEPDC5
PIK3CA
DPYD
EGFR
BRCA1

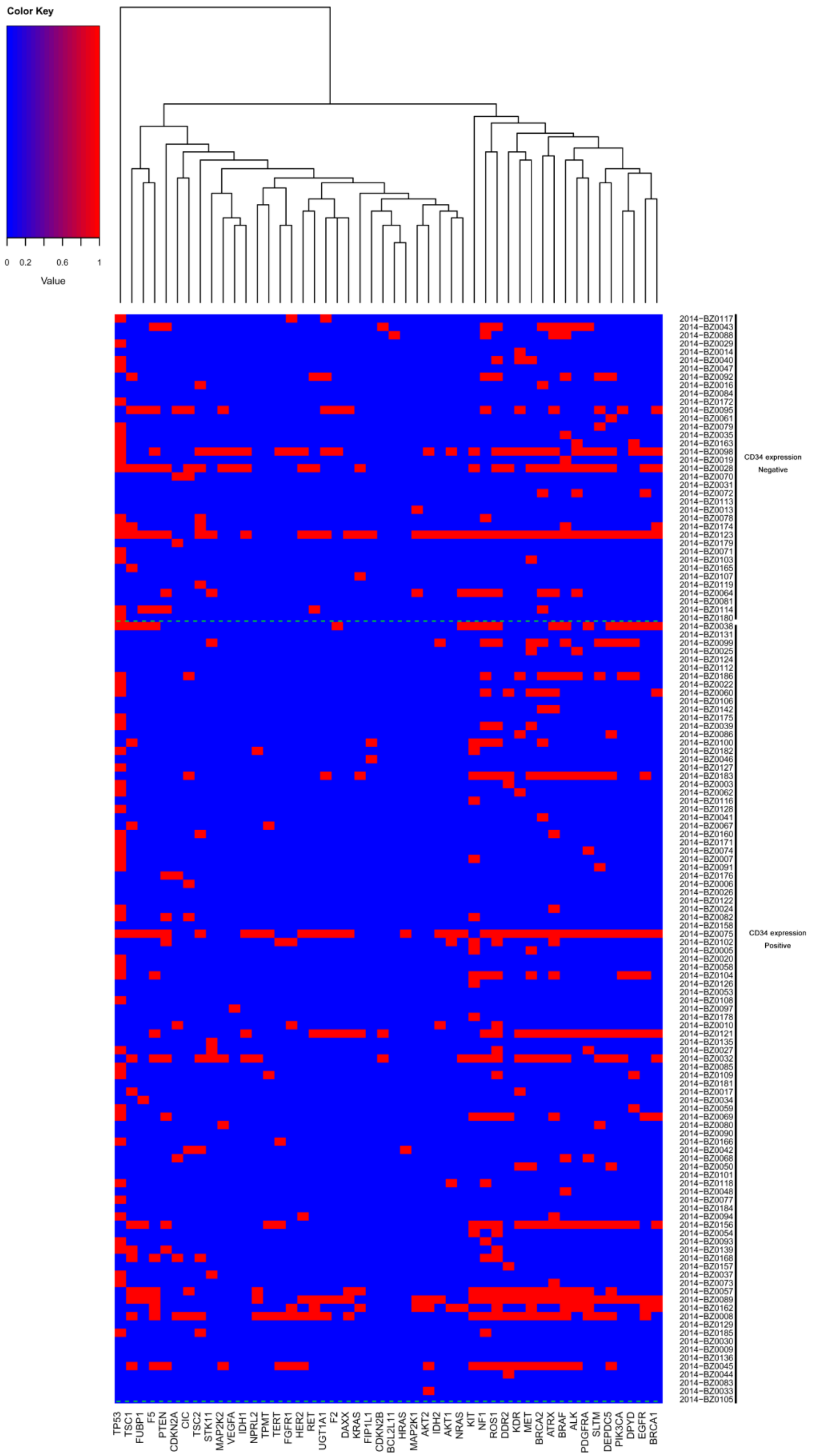

Supplementary Figure S5-S13: Variation profile of 48 genes based on clinicopathological features. Hierarchical cluster analysis result for all patients (S5), and stratified cluster analysis indicated that no obvious differences of variation profile were found between sex (S6), age (S7), tumor sites (S8), tumor sizes (S9), mitosis (S10), CD117 (S11), DOG-1 (S12), and CD34 (S13) expressions.
